# Supplementary material for: High sensitivity of the Bionote Anigen Rapid Rabies Antigen Test in detection of diverse rabies virus variants suggests utility for global rabies control
Source: J Clin Microbiol. 2026 May 22;64(6):e01605-25. doi: 10.1128/jcm.01605-25 (PMC13251392; doi:10.1128/jcm.01605-25)
Supplement: Supplemental materials — Modified protocol of the BioNote Anigen Lateral Flow Assay. [file jcm.01605-25-s0001.docx]

# Supplementary Figures and Tables

**Appendix A**

**BIONOTE Rabies Anigen Test Kit®** **Study Comparison of Lateral Flow Assay (BN-LFA) (CDC Quick Reference Protocol) and Direct Fluorescent Antibody Test [30]** provided to all participating laboratories

**Introduction**

**General safety**

All rabies diagnostic procedures should be performed following biosafety precautions for handling Lyssaviruses and following the laboratory’s site-specific risk assessment including review of the relevant best practices in the current Biosafety in Microbiological and Biomedical Laboratories- 6^th^ Edition (BMBL). Prior to start of testing, risk assessments should account for the vaccination status of laboratory personnel providing testing (pre-exposure rabies immunization and monitoring of titer), as well as the protocols (brain collection, test protocols, waste disposal and decontamination) and the PPE required at each step.  The minimum personal protective equipment (PPE) required for Lateral Flow Assay (LFA) testing after brain removal includes: lab coat, safety glasses, double gloves and N95 mask; face shields are optional. These biosafety precautions adequately address all reasonable rabies virus exposure concerns. If a BSC is used, then N95 mask, safety glasses and face shield are not required.) If there are variations in these recommendations, additional steps can be taken to mitigate the risk of rabies virus exposure; a site-specific risk assessment should be performed to determine additional mitigations that may be necessary.

All reusable materials and equipment used in testing should be decontaminated using a recommended disinfectant (1:256 QAC, 10% bleach, 10% iodophor) after use, and disposable materials should be incinerated or autoclaved before discard. Personnel should be aware of the potential hazards and how to avoid or reduce these hazards prior to initiating rabies testing (WHO Laboratory Techniques in Rabies Chapter Biosafety 2018, BMBL 2020).  If the Lateral Flow Assay is to be performed in a laboratory and a Class II BSC is available, then it should be used for this testing. However, the BioNote Rabies Anigen Test Kit was designed to be a rapid field test and can be performed easily and safely if the aforementioned biosafety recommendations are taken. If this protocol is modified in a manner that may result in aerosolization of rabies virus, additional biosafety actions should be considered.

Supplemental Figure 1


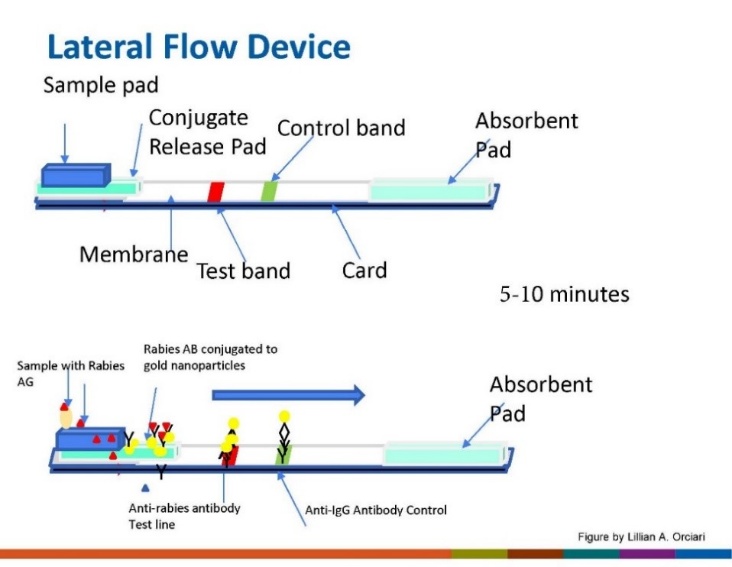


**Principle of the test**

Lateral flow assays, which are more formally known as rabies immunochromatographic diagnostic tests (RIDC), are most frequently used to detect rabies virus antigen. A brain suspension is added to the opening in the device cassette and is adsorbed into the sample pad (Supplemental Figure 1). The sample then flows through the conjugate pad containing anti-rabies antibodies labeled with gold nanoparticles.

If rabies virus antigen is present, nanoparticle-labeled antigen-antibody complex diffuses down the membrane strip and reacts with the test band containing a second anti-rabies antibody and a colored band appears (Supplemental Figure 2). When the suspension flows to the control band, the anti-IgG will react with the anti-rabies antibodies and the control band will appear. For the test to be valid, the reagents would need to flow appropriately along the membrane strip, and the conjugate labeled with nanoparticles (either bound with antigen or unbound in case of a negative sample) must react with the anti-IgG control band.

*Supplemental Figure 2.*


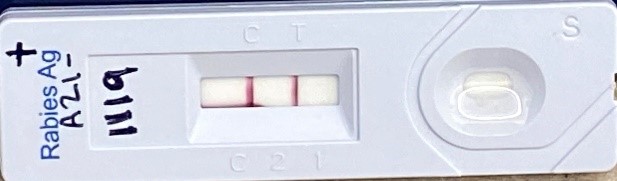

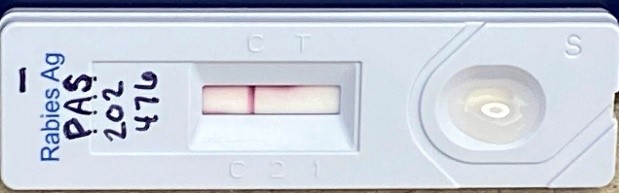


*Positive BN-LFA Test                                                                         Negative BN-LFA Test*

While the concept of these assays is good, in reality LFAs have not performed as well as expected (lower sensitivity and specificity) when used according to the manufacturers’ recommendations. CDC is currently working on a method to improve the sensitivity of the LFAs for rabies antigen detection and will be working with the collaborators in the USA as well as international partners to test more thoroughly with the modifications proposed here.

**Brain Collection**

The brain sample (brain stem, full cross-section) is collected for the LFA using the same standard operating procedure and PPE as for collection of brain stem for DFA or DRIT. This sample can be taken from dorsal side or ventral side via the foramen magnum using a scalpel or spatula as well as from a fully excised brain sample. For this study, a full cross-section of brain stem must be tested, and sufficient tissue collected for LFA, DFA or DRIT, and confirmatory testing at a reference laboratory in the case of discordant findings. To ensure the identical cross-section of brain stem is tested by both LFA and DFA or DRIT, harvest a single cross-sectional piece of brain stem for the tests required and place it in a container so that the orientation can be easily identified. With care, immediately make a cross-sectional slice through the brain stem piece for LFA and place it in a separate container. Repeat this process making adjacent cross-sectional slices for DFA or DRIT and confirmatory testing.

**DO NOT USE THE STRAW COLLECTION METHOD.** Brain tissues cannot be easily identified when extracted using the straw, especially tubular pieces extracted of neocortex (white tissues from the cerebral cortex) cannot be easily distinguished from brain stem. Depending on the size of the animal, the straw method may not provide full cross-section of the brain stem, impacting interpretability of the test.

**LFA method modified by CDC**

*Supplemental Figure 3*


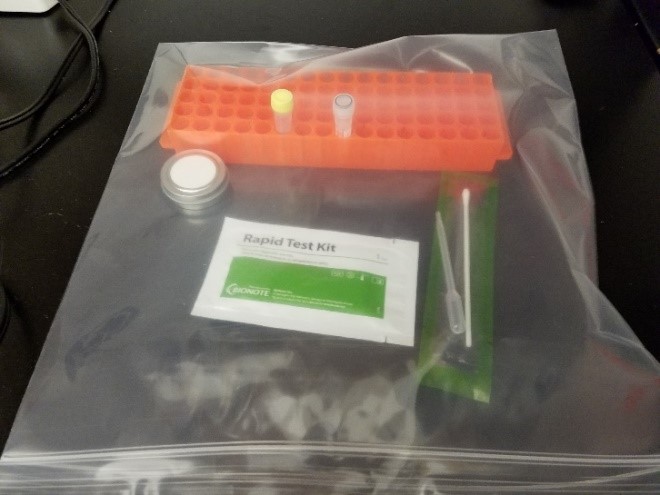


The BioNote “Rabies Anigen Test Kit” was designed to be a rapid field test and can be performed easily and safely in the field providing additional containment steps are performed to avoid or reduce potential risks. If the test can be performed in a laboratory with a BSC then the additional containment options are not necessary. If the aforementioned biosafety measures are followed, no additional biocontainment is necessary for using the LFA in the field (Option 1). If there are deviations in the biosafety procedures that may result in aerosolization of brain homogenate, then additional biocontainment options should be considered (Option 2).

Option 1: Part of the procedure is performed outside of the bag.  
Option 2: The entire LFA protocol may be performed inside of a 1 gallon size (12”x12”x12”) plastic Ziplock bag (Supplemental Figure 3).

**Materials needed (Not provided in the BioNote Test Kit)**

- Gallon size Ziplock bags (optional, based on biosafety assessment)
- 2 ml Sarstedt tubes (screw cap tubes equipped
- Autoclavable plastic test tube rack for 2 ml Sarstedt tubes
- Sharps container for scalpels and other sharps
- Scalpels
- Discard container with disinfectant (e.g. 1:256 QAC, 10% bleach, 10% iodophor)
- PPE (lab coat, N95 mask, gloves, safety glasses and/or face shield)
- Permanent fine tip marker
- Lab mat
- Autoclave bag for disposable material

**Materials needed** (Provided in the BioNote Test)

- Anigen Rapid Rabies Ag Test Devices
- Assay diluent tubes
- Disposable swabs
- Disposable droppers

**CDC SOP**

Allow reagents to come to room temperature prior to use.

If a BSC is not available or biosafety recommendations cannot be fully adhered to, place a lab mat on a level bench surface and a gallon size Ziplock bag containing, the test tube rack with an empty Sarstedt tube and assay diluent tube, 1 swab and Anigen Rapid Rabies Ag Test Device. Within the BSC or inside large Ziplock bag arrange the needed materials (Supplemental Figure 3 from previous page).

*Supplemental Figure 4*


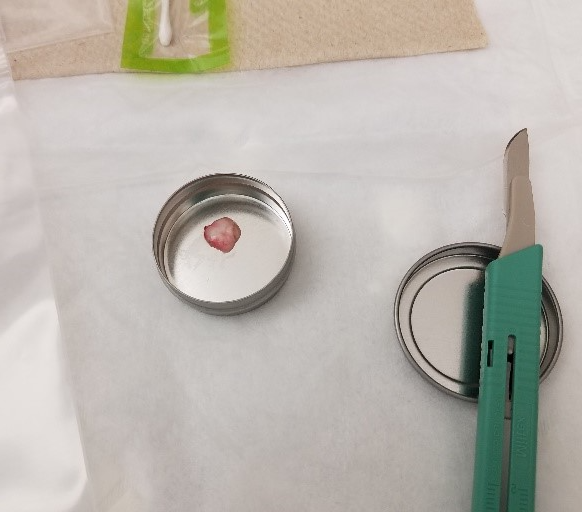


Cross-section of

Brain Stem

1. Take a piece of brain stem (full cross-section) usually (0.1 g to 0.2 g), the size of the (BioNote) cotton tip (Supplemental Figure 4). If the brain stem is from a large animal the full cross section should still be collected.

*Supplemental Figure 5 (a and b)*

 

b

a

1. Mince tissue as finely as possible (to paste Supplemental Figure 5a) with a scalpel (best to have tin securely on the bench while cutting tissue to avoid potential sharps injury with scalpel (held in photo to get best angle for demonstration and pictures) and transfer the tissue to 2 ml Sarstedt tube or 15 ml centrifuge tube for large animals used for coating the swab (Supplemental Figure 5b). It would be safer and preferrable to transfer the minced tissue to the tube with a swab rather than the scalpel as shown here.

*Supplemental Figure 6.*

1. With the swab provided in the kit (Supplemental Figure 6), rub the brain tissue against the inside of the tube with the swab until the brain consistency is a smooth paste and the swab is coated with brain (Figure 6). This process may up to 1 minute or more depending on how well the brain was minced in the previous step. The tube should face away from any personnel in the vicinity, to avoid unintentional splashes.
2. Transfer the swab coated with the brain from the Sarstedt tube to the assay diluent tube. (There should be no chunks of tissue, resulting from brain that was not fully minced to a paste).

*Supplemental Figure 7*


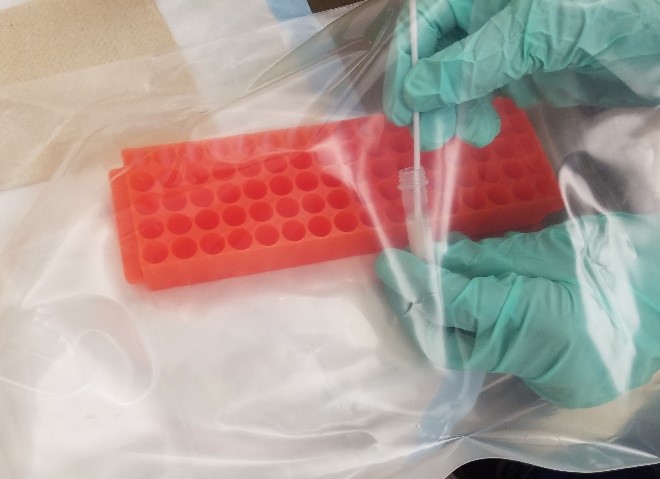


1. Prepare an even suspension in the assay diluent tube by rubbing the swab coated with brain paste gently against the inside of the tube for at least 10 seconds until brain is completely blended into the diluent, it may take longer (*Supplemental Figure 7*). 
    
   *Supplemental Figure 7 (note, use of Ziploc bag is optional, based on adherence to recommended biosafety measures for field use when a BSC is not available).*
2. If a bag is used, carefully open just prior to applying the sample. Label the LFA with a permanent marker (date, sample #, initials of operator).

*Supplemental Figure 8*


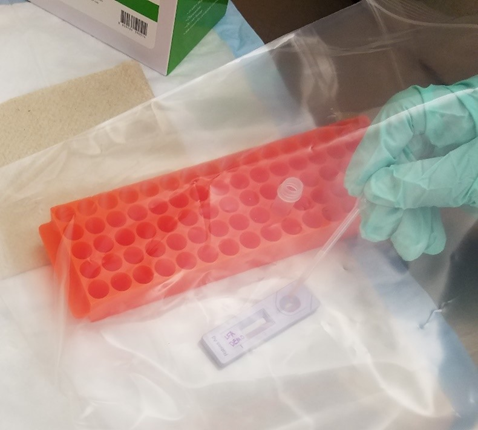


1. Add 4 drops of the sample to the well on the LFA with the disposable dropper provided in the BioNote kit. Set timer for 10 minutes (*Supplemental Figure 8*). 
    
   *Supplemental Figure 8 (note, use of Ziploc bag is optional, based on adherence to recommended biosafety measures for field use when a BSC is unavailable).*

*Supplemental Figure 9*

 
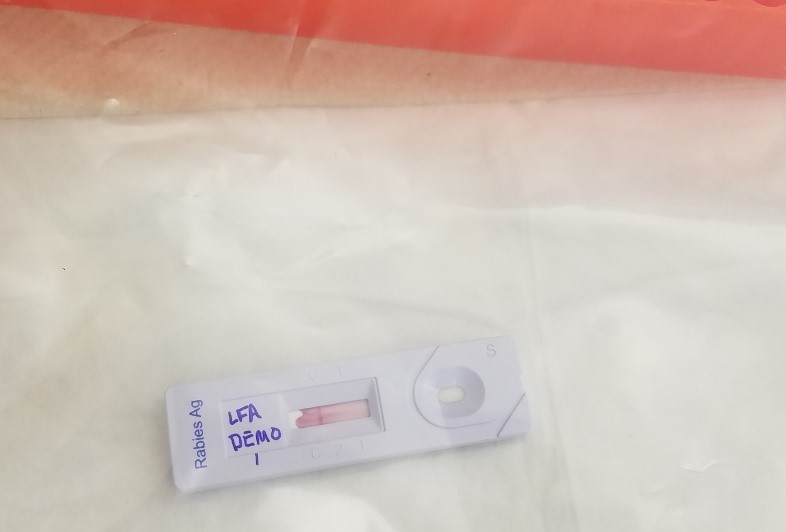


1. Movement of the sample liquid into the secondary chamber of the LFA should be noticed in about one minute. If movement of sample is not noticed add another drop (only one) of the sample (Figure 9).

*Supplemental Figure 9 (note, use of Ziploc bag is optional, based on adherence to recommended biosafety measures for field use).*

1. Record results (on the device and worksheet) once the band(s) are apparent in 5-10 minutes. After 10 minutes the reactions are complete and final. Reading results after 10 minutes could potentially lead to false positive results (Figure 10), however it has not been noticed in these studies.

*Supplemental Figure 10.*


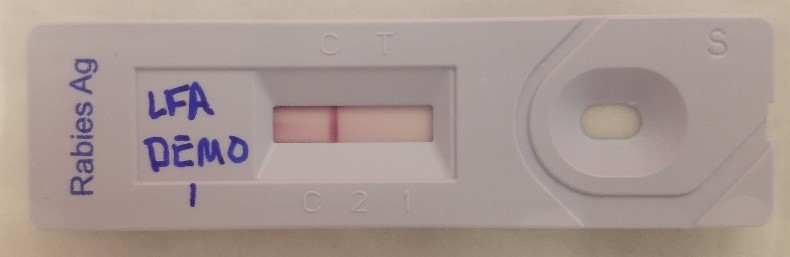


*LFA Demo sample is negative after 10 minutes (Control band and no test band)*

1. Take a photograph at 10 minutes incubation for a permanent record.

**Interpretation of results**

1. Negative results: C (Control) band only, Positive results C and T (test) bands present, and invalid results no C band present (*Supplemental Figure 11*).

*Supplemental Figure 11.*


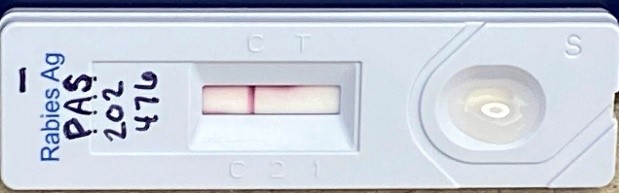

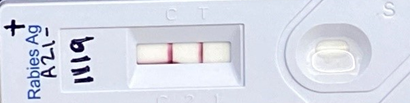


*Negative Test                                                                Positive Test*

 
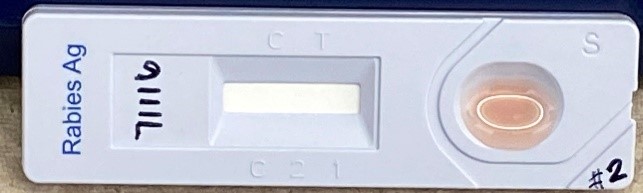


*Invalid Test*

1. Invalid results should be repeated immediately using another LFA kit. If repeat test is invalid, test will be reported as invalid.

1. Discordant results with the DFA or DRIT can also be repeated using this same procedure. Do not replace the original results in the tracking sheet, rather start a new record that clearly denotes that this is a repeat test based on discordant results.

**Decontamination and clean up**

1. Tubes swabs and LFA can be placed in a container of appropriate disinfectant and autoclaved, lab mat and other disposable material may be autoclaved or incinerated prior discard.

1. Work surfaces and non-disposable equipment used for testing should be decontaminated using an approved disinfectant after testing is completed.
